# The Effects of *Bacillus amyloliquefaciens* SC06 on Behavior and Brain Function in Broilers Infected by *Clostridium perfringens*

**DOI:** 10.3390/ani14111547

**Published:** 2024-05-23

**Authors:** Siyu Chen, Jinling Liu, Shuyan Luo, Limin Xing, Weifen Li, Li Gong

**Affiliations:** 1 Guangdong Provincial Key Laboratory of Animal Molecular Design and Precise Breeding, School of Life Science and Engineering, Foshan University, Foshan 528250, China; chensiyu@fosu.edu.cn (S.C.); 13074344623@163.com (J.L.); shuyenlo@163.com (S.L.); xlm1592584344@163.com (L.X.); 2 Key Laboratory of Animal Feed and Nutrition of Zhejiang Province, Institute of Animal Nutrition and Feed Sciences, College of Animal Sciences, Zhejiang University, Hangzhou 310058, China

**Keywords:** *Bacillus amyloliquefaciens* SC06, broiler, behaviors, mRNA sequence, brain functions, *Clostridium perfringens*

## Abstract

**Simple Summary:**

Necrotic enteritis caused by *Clostridium perfringens* (CP) is an important disease for the poultry industry. It leads to decreased growth and production performance in chickens. *Bacillus amyloliquefaciens SC06* (BaSC06) is known to play a role in preventing damage from the bacterial infection. However, whether CP could affect brain function and behavior and whether BaSC06 has a preventive role in infected animals are not yet reported. Thus, the aim of this study was to investigate whether BaSC06 has a preventative effect on broiler chickens infected by CP. Our results showed that CP is associated with a reduction in stress and fear-related behaviors and causes pathological damage to the pia and cortex of the brain, as well as inhibiting the expression of genes related to stress, while the treatment of BaSC06 alleviates these adverse effects.

**Abstract:**

Poultry studies conducted on *Clostridium perfringens* (CP) mainly focus on the effects of intestinal health and productive performance. Notably, the probiotic *Bacillus amyloliquefaciens SC06* (BaSC06) is known to play a role in preventing bacterial infection. However, whether CP could induce the changes in brain function and behaviors and whether BaSC06 could play roles in these parameters is yet to be reported. The aim of this study was to evaluate the effects of BaSC06 on stress-related behaviors and gene expression, as well as the brain morphology and mRNA sequence of the hypothalamus in broiler chickens. A total of 288 one-day-old chicks were randomly divided into four groups: (1) a control group with no treatment administered or infection; (2) birds treated with the BaSC06 group; (3) a CP group; and (4) a BaSC06 plus CP (Ba_CP) group. The results showed that stress and fear-related behaviors were significantly induced by a CP infection and decreased due to the treatment of BaSC06. CP infection caused pathological damage to the pia and cortex of the brain, while BaSC06 showed a protective effect. CP significantly inhibited hypothalamic *GABA* and promoted *HTR1A* gene expression, while BaSC06 promoted *GABA* and decreased *HTR1A* gene expression. The different genes were nearly found between the comparisons of control vs. Ba group and Ba vs. CP group, while there were a great number of different genes between the comparisons of control vs. Ba_CP as well as CP vs. Ba_CP. Several different gene expression pathways were found that were related to disease, energy metabolism, and nervous system development. Our results will help to promote poultry welfare and health, as well as provide insights into probiotics to replace antibiotics and reduce resistance in the chicken industry.

## 1. Introduction

*Clostridium perfringens* (CP) is an anaerobic zoonotic pathogenic bacterium with a positive Gram stain [1]. CP widely exists in the intestines of humans and animals, as well as in vegetation, rivers, and soil. It is a common disease in various natural environments [2], and it is one of the causes of both chronic and acute necrotic enteritis (NE) disease in poultry. Chronic NE can cause loss of appetite, reduced intestinal absorption, a lowered feed conversion rate, and weight loss, leading to a reduction in poultry performance. Acute NE can cause sudden and massive death of poultry, with features such as inflammation, multifocal, irregular, plaque-like mucosal ulcers, and necrosis in the upper part of the small intestine after dissection [3,4,5].

To date, poultry studies conducted on CP mainly focus on the effects of intestinal health. In broilers, the subclinical form of chronic NE is currently the common symptom. Due to the difficulty of being detected and treated in a timely manner during production, chronic NE is the main cause of economic losses [6]. Different strains of CP have different effects on intestinal integrity, immunity, mucus production, and nutrient transport proteins by regulating intestinal genes encoding proteins responsible for apoptosis [7]. The infection of CP type A induces intestinal inflammation, which may be mediated by Th2 and Th17 cells in broilers [8], and generally, NE is mainly caused by CP type A, with very few cases being caused by type C in broilers [9]. A previous study reported that *Scutellaria baicalensis and Lonicerae Flos* extract could effectively mitigate the negative effects of CP challenge by improving intestinal barrier function and histomorphology, positively influencing the growth performance of Chinese yellow-feather broilers [10]. Recent studies have shown that the enterotoxin of CP can cross the blood-brain barrier [11]. CP type A can produce a large amount of alpha toxins, which are one of the main toxins that cause NE. The alpha toxin of CP type A can disrupt the target cell membrane by hydrolyzing the components of the host cell membrane, leading to endocytosis and cell death [12,13,14]. In addition, the alpha toxin of CP type A can breach the blood-brain barrier and invade the neuronal cytoplasm of the medulla oblongata [11].

Probiotics are live microorganisms that exert their benefits on the host when administered in adequate amounts [15]. Diets supplemented with *Bacillus* is known to reduce the frequency of aggressive behavior in layers [16], and *Bacillus coagulans* has been shown to prevent cognitive decline and attenuate hippocampal damage in mice [17]. *Bacillus subtilis* improved heat stress-related behaviors and immune responses by modulating the microbiome and immunity [18]. Further, probiotics have been shown to effectively treat symptoms of drug-resistant depression [19,20]. The possible mechanism of probiotics on behavior is argued to be modulated through the role of the gut-brain axis [21,22,23]. In this study, we aimed to investigate the effects of administering BaSC06 in response to a CP infection on broiler behavior and brain function. The finding would help improve broiler welfare and health and provide new insights for the sustainable development of the poultry industry.

## 2. Materials and Methods

### 2.1. Experimental Animals and Design

This study was approved by the Animal Care Committee of Foshan University (Approval ID: FOSU#119). Male broilers, a hybrid called 817 from white-feathered broiler and layers, with an initial average body weight of 36.15 ± 0.1 g, were purchased from Guangzhou Muyuan Poultry Co., LTD (Guangzhou, China). They were brooded in a facility at Foshan University, Foshan City, China, where there was a three-tiered battery cage with 0.12 m^2^ per bird. The temperature was kept above 32 °C from post-hatching to 16 days old. Thereafter, the temperature was gradually decreased to room temperature at the age of 50 days (slaughter day). A total of 288 birds were randomly divided into four groups, with six replicates per group and 12 birds per replicate. They were the control group (control, n = 72), a *Bacillus amyloliquefaciens* SC06 group (Ba, n = 72), a *Clostridium perfringens* group (CP, n = 72), and a *Bacillus amyloliquefaciens* SC06 plus *Clostridium perfringens* group (Ba_CP, n = 72). All birds were fed the same diet, a commercial feed ration, while birds in Ba and Ba_CP were also provided with BaSC06 (5 × 10^8^ cfu·kg^−1^ feed). The dosage was based on group members and a preliminary experiment. The main contents of the diet are described in Table S1, and the BaSC06 treatment was prepared by the Microbiology and Genetic Engineering Laboratory, Institute of Animal Science, Zhejiang University. On the 17th, 18th, 19th, and 20th days of age, chickens in the CP and Ba_CP groups were fed 1.5 mL 4 × 10^8^ CFU/mL CP type A (CVCC2030) bacterial solution to infect the CP disease, which was purchased from the China Veterinary Microbial Species Conservation Management Center. Birds in the challenged groups were orally challenged with an actively growing *C. perfringens* type A strain, while the non-challenged birds were treated with the same volume of sterilized Reinforced Clostridium medium.

### 2.2. Behavioral Traits

An open field test is a common measure of exploratory behavior, general activity, and fear to indicate stress levels in both laboratory and farm animals, where both the quality and quantity of the activity can be measured [24]. The day before the test, chickens were deprived of food and water from 18:00 and individually identifiable by colored leg rings. At 37 and 38 days of age, 15 birds in each group were randomly moved to a test arena (length × width × height: 240 cm × 180 cm × 100 cm) enclosed by solid panels. Feed was placed in one of the corners. Behaviors including exploration, feeding, flapping, stand-resting, aggression, feather pecking, lay-resting, and preening (the definition of behaviors can be found in Table S2) were observed by continuous observation of videos for 10 min. A single observer extracted the data from the videos.

A vigilance test was used to quantify the fear and stress levels, which was applied to many farm animals [25]. Fifteen chickens from each group used in the novel arena test were placed in the same arena to test vigilance in response to a predator immediately after the open field test. When tested, regular feed was placed in one corner of the arena, where a hawk model (length 30 cm and width 30 cm) was placed 50 cm vertically above the feed. Furthermore, the calling of a hawk was played three times (at 4, 8, and 12 min), during the 12-min test. The reaction of the individual was scored on a scale from 0 to 4 using a model from previous studies [25,26], where 0 represented the lowest fear response. A score of 0 represented no visible change in the chicken’s behavior; 1 was scored if the bird lifted its head once and then immediately returned to exploration or eating; 2 was scored if the chicken lifted its head once and uttered an alarm call and/or walked rapidly for >3 s or froze for 3 to 20 s; score 3 represented if the bird reacted as for score 2 but ran, attempted to escape, or froze for > 20 s. The duration of the freezing time and behaviors, including stand-resting, lethargy, crouching, and lay-resting, were observed.

### 2.3. Hematoxylin–Eosin (HE) Staining 

At 21 and 50 days of age, six chickens from each group were randomly selected and humanely slaughtered by rapid decapitation. The left hemisphere of the brain of four birds in each group was collected and immersed in 4% paraformaldehyde fixating solution (Shenzhen Xijing Biotechnology Co., LTD. (Shenzhen, China)) to make tissue slices. Then, HE staining was performed.

### 2.4. Transcriptome Sequencing

At day 50, six birds were euthanized, and the hypothalamus was collected. The samples were immediately stored in liquid nitrogen after collection. Total RNA isolation was performed using TRIzol reagent (Invitrogen, Carlsbad, CA, USA). Transcriptomic data were analyzed by the support of Gidio Biotechnology Co., LTD., Guangzhou, China. The library construction was prepared using NEBNext Ultra RNA Library Prep Kit for Illumina (NEB, Beverly, MA, USA), and an Illumina Hiseq platform was used to generate paired-end 150 bp reads.

Fastp was used to perform quality control on raw reads. Then, the accurate reads were compared to the chicken rRNA database using ‘bowtie2’, and the corresponding reads were removed. HISAT2 [27](Kim et al., 2015) was then used to compare the spliced reads and reads of different lengths to the chicken genome by different calculation methods (Gallus_Gallus-5.0). DESeq2 [28] (Love et al., 2014) software was used for standardization. Finally, the false discovery rate (FDR) was obtained by multiple hypothesis testing, and FDR < 0.05 and log2 (Fold Change) >1 were screened as significant differential genes.

### 2.5. Gene Expressions

The hypothalamuses were further used for the determination of gene expression. High-quality total RNA was isolated using the RNeasy Mini-Extraction kit (Aidlab, RN2802, Beijing, China), according to the manufacturer’s protocols. Then, qRT-PCR was performed to determine the expression of stress-related genes including 5-hydroxytryptamine receptor 1A (*HTR1A*), dopamine (*DA*), Gamma-aminobutyric acid (*GABA*), and Gamma-aminobutyric acid type A receptor subunit beta1 (*GABRB1*) in the hypothalamus using the ABI 7500 Realtime Detection System (Applied Biosystems, Massachusetts, MA, USA) and RTPCR reagents (TransGen Biotech, Beijing, China). Each 20 μL PCR reaction system contained 10 μL of 2 × TransStart Top/Tip Green Qpce, 0.4 μL (10 pM) of each primer, 0.4 μL of Passive Reference Dye (50×), 0.8 μL of cDNA (100 ng), and 8 μL of ddH_2_O. After an initial denaturing for 30 s at 95 °C, there were 40 cycles of amplification (95 °C for 15 s, 57 °C for 30 s, and 72 °C for 85 s), followed by thermal denaturing to generate melting curves. *GAPDH* was amplified in the same plates as endogenous controls. Samples were assayed in triplicate for standard curves. PCR efficiency, amplification efficiency of the transcripts of interest, and the internal standard of *GAPDH* were consistent with the measurement of the above genes. Dissociation curves verified that the amplification was specific. Relative quantitative expression of the target gene was calculated using the 2^−ΔΔCt^ method [29] (Livak and Schmittgen, 2001). The primers of stress-related genes and *GAPDH* are shown in Supplemental Material Table S3.

Six differentially expressed genes, including three up- and three down-regulated genes, were selected to verify the accuracy of the sequencing data using qRT-PCR. The primers for these selected genes are shown in Supplemental Material Table S3.

### 2.6. Statistical Analysis

The data were analyzed by IBM SPSS 22.0. Gene expressions were checked for normality and homogeneity of variance test and analyzed using a one-way ANOVA. The behavioral data did not meet the assumptions for parametric analysis, and the Kruskal–Walli’s test was therefore conducted. A relative to an identified distribution (Ridit) analysis was used to assess the fear score in response to the vigilance test. All values with *p* < 0.05 were regarded as statistically significant.

## 3. Results

### 3.1. Behavioral Traits

**Figure 1:**
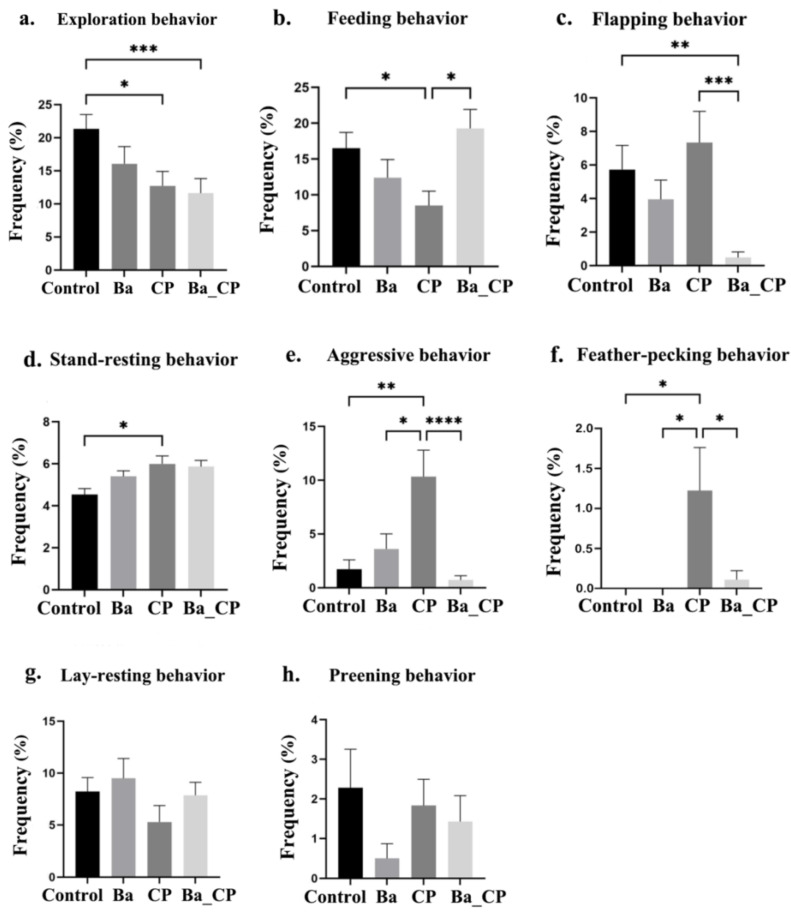
Frequency of behavior in the open field test. (**a**) Exploration behavior. (**b**) Feeding behavior. (**c**) Flapping behavior. (**d**) Stand-resting behavior. (**e**) Aggressive behavior. (**f**) Feather-pecking behavior. (**g**) Lay-resting behavior. (**h**) Preening behavior. *, **, ***, **** indicates significant difference between the two groups with the value of *p* < 0.05, *p* < 0.01, *p* < 0.001, and *p* < 0.0001, respectively.

In response to the open field test, the exploration behavior of the control group was significantly higher than that of the CP group (*p* < 0.05) and Ba_CP (*p* < 0.001) groups (Figure 1a). Feeding behavior was significantly lower in the CP group compared to the control group, and increased in chickens that had received treatment with BaSC06 (*p* < 0.05, Figure 1b). Flapping behavior was significantly increased after CP infection (*p* < 0.01, Figure 1c), but was significantly lower in the Ba group (*p* < 0.001, Figure 1c). Stand-resting behavior was increased after the administration of CP compared to the control group (*p* < 0.05, Figure 1d). Aggressive behavior and feather-pecking behavior were both increased in the CP group, and both were reduced by the treatment of BaSC06 (*p* < 0.05, Figure 1e,f). Lay-resting and preening behaviors were not different among groups.

**Figure 2:**
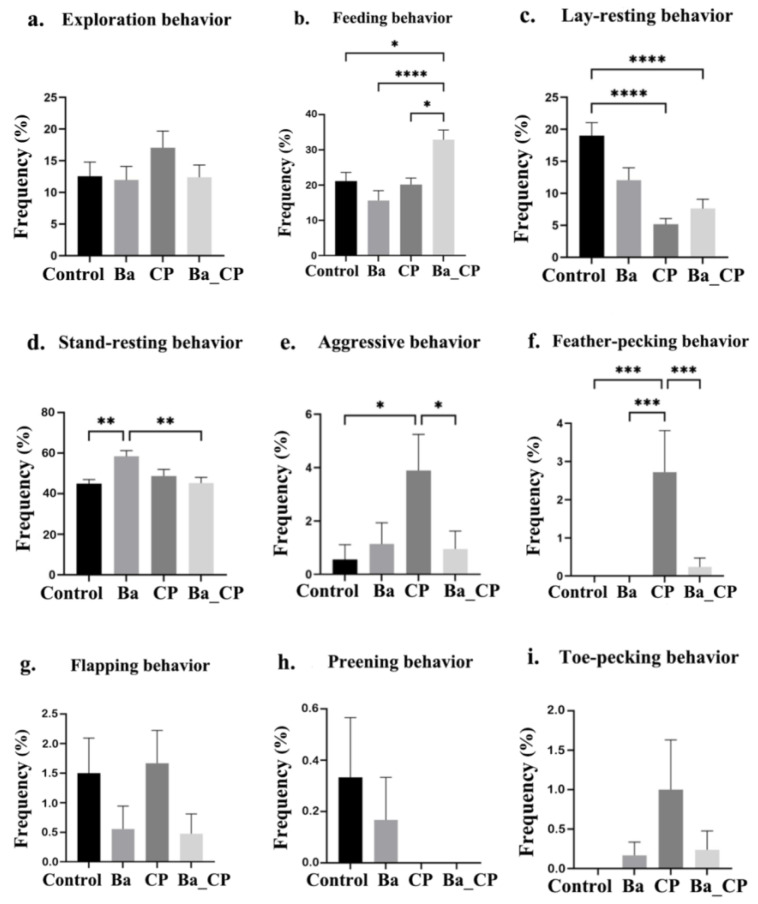
The frequency of behaviors in vigilance test: (**a**) exploration behavior; (**b**) feeding behavior; (**c**) lay-resting behavior; (**d**) stand-resting behavior; (**e**) aggressive behavior; (**f**) feather-pecking behavior; (**g**) flapping behavior; (**h**) preening behavior; and (**i**) toe-pecking behavior. *, **, ***, **** indicates significant difference between the two groups with the value of *p* < 0.05, *p* < 0.01, *p* < 0.001, and *p* < 0.0001, respectively.

In the vigilance test (Figure 2), exploration behavior was not different among the groups (Figure 2a). Feeding behavior was increased in the Ba group compared to the control and CP groups (*p* < 0.05, Figure 2b). The lay-resting behavior was significantly higher in the control group than in the CP and Ba_CP groups (*p* < 0.001, Figure 2c). Stand-resting behavior was induced in the group with BaSC06 treatment (*p* < 0.01, Figure 2d). The aggressive behavior and feather-pecking behaviors were both higher in the CP group and decreased by the treatment of BaSC06 (*p* < 0.05). Toe-pecking behavior in the CP group was significantly higher than that in the control group, Ba group, and Ba_CP groups (*p* < 0.001, Figure 2e,f). Flapping, grooming, and toe-pecking behaviors were not significantly different among groups.

**Figure 3:**
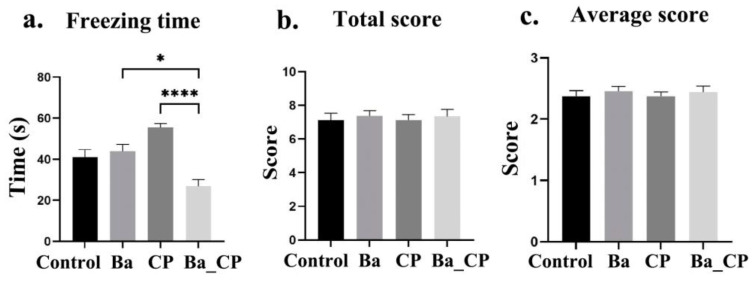
Freeze behavior: (**a**) freezing time; (**b**) total score; and (**c**) average score. *, **** indicates significant difference between the two groups with the value of *p* < 0.05 and *p* < 0.0001, respectively.

Freezing time was significantly increased by CP infection, and reduced in the Ba group (*p* < 0.05, Figure 3). The total score and the average score were not different among groups.

### 3.2. Gene Expression

**Figure 4:**
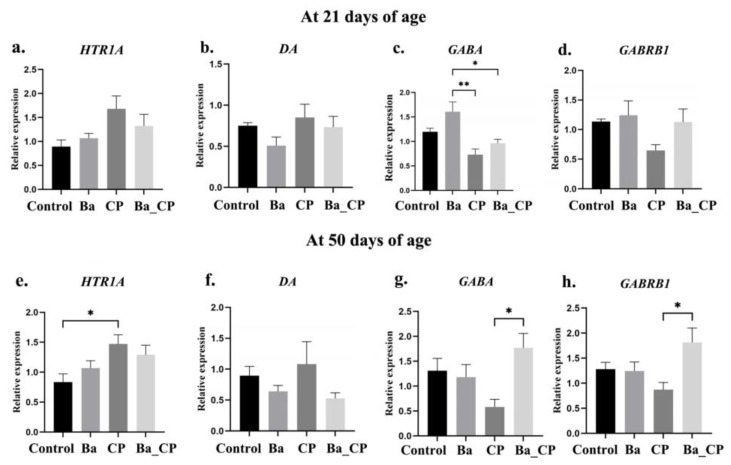
Relative expression levels of stress-behavior-related genes in the hypothalamus of broilers. (**a**,**e**) were *HTR1A*; (**b**,**f**) were *DA*; (**c**,**g**) were *GABA*; and (**d**,**h**) were *GABRB1*. *, ** indicates significant difference between the two groups with the value of *p* < 0.05 and *p* < 0.01, respectively.

At 21 days of age, the relative expression of the *GABA* gene in the Ba group was significantly higher than in the CP group (*p* < 0.01) and Ba_CP group (*p* < 0.05) (Figure 4a). The relative expression of *HTR1A*, *DA* and *GABRB1* were not statistically different among groups (Figure 4a–c).

At 50 days of age, the relative expression of *HTR1*A was significantly increased in the CP group compared to the control group (*p* < 0.05, Figure 4e). The relative expression of *GABA* and *GABRB1* were increased in the Ba_CP compared to the CP group (*p* < 0.05, Figure 4g,h).

### 3.3. Hematoxylin–Eosin Staining

At 21 days of age, the brain leptomeninges in the CP group were thickened (Figure S1g), some neurons in the cerebral cortex were deeply stained, and the structure of neurons was unclear (Figure S1h). Some neurons presented with edema, and the cells were vacuolated (Figure S1h). The structure of the cerebral cortex was loose, the brain tissue had focal necrosis, and the neuron cells were reduced in size (Figure S1i). The leptomeninges in the Ba_CP group were not thickened, the cerebral cortex was tightly structured, and the nucleus was clearly visible (Figure S1j–l).

At 50 days of age, the leptomeninges in the CP group were loose and thickened (Figure S2g), and microvascular hemorrhage was observed in the cerebral cortex, especially around the blood vessels, with vascular dilation, edema, and a vascular sheath phenomenon (Figure S2h). The number of vascular sheaths increased (Figure S2h), and the cerebral cortex structure was loose and sparsely arranged, accompanied by mild edema. The structure of neurons was not clear (Figure S2i). The leptomeninges in the Ba_CP group were not thickened, and the structure of the meninges was looser compared with those in the CP group (Figure S2j). In the CP group, the structure of the cerebral cortex was tight, the nucleus was clearly visible, and no vascular sheath was formed (Figure S2k,l).

### 3.4. mRNA Sequencing

The quality of the sequencing of data is shown in Tables S4 and S5. The two commonly used indicators for sequencing had a Q30 value that was between 92.31% and 93.65%. The GC content ranged from 46.04 to 47.68, both of which were at the standard level, and the indicators were consistent across samples. In the rRNA comparison, the average number of remaining reads after rRNA filtering was 40.17 million. The genome comparison results showed that 93.39–94.83% of effective reads were located in the chicken reference genome. The regional proportion was 73.72–94.12%.

The relative expression of genes was not different between the control and Ba groups. In the comparison between the control and CP groups, there were four significantly up-regulated and two significantly down-regulated genes. There were 5819 significantly different genes between the control and Ba_CP groups, including 5547 significantly up-regulated and 272 significantly down-regulated genes. In the comparison between the Ba and CP groups, there was only one significantly up- and one down-regulated gene. Between the Ba and Ba_CP groups, there were 6136 significantly different genes, including 5820 up-regulated and 316 down-regulated genes. In the comparison between the CP and Ba_CP groups, there were 3549 significantly different genes, including 3170 significantly up- and 379 significantly down-regulated genes. The top five up- and down-regulated genes are shown in Table 1.

In the control vs. CP groups, the differentially expressed genes were significantly enriched into three pathways (Table 1). There were 349 significantly enriched pathways between the control and Ba_CP groups. The top 10 pathways were the longevity regulating pathway, mammal, synthesis, secretion, and function of growth hormone synthesis, secretion, and action pathways (Table 1). Between the Ba and CP groups, there were 19 significantly enriched pathways. The top 10 pathways were graft-versus-host disease, viral myocarditis, and viral carcinogenesis. In the comparison of group Ba vs. Ba_CP, 345 pathways were differently enriched; the top pathways were animal autophagy, axon orientation, gonadal hormone secretion, and gndocytosis. Between CP and Ba_CP, there were 339 significantly enriched pathways, the top five of which were cholinergic synapse, insulin secretion function index, growth hormone synthesis, secretion and action, and morphine addiction.

The gene expression of RT-qPCR was consistent with that of mRNA-sequencing, which proved the reliability of the sequencing data in this study (Figure S3).

## 4. Discussion

Animals typically display anxiety-like and fearful behaviors and exhibit fewer exploratory behaviors when they encounter novel environments. This is called self-protecting behavior [30]. In this study, we found that birds infected with CP showed less exploratory behavior and more standing behavior compared to control birds, suggesting a stressful state and that these birds may be reluctant to explore a novel environment [31]. A previous study indicated that fear- and anxiety-like behaviors resulted in reduced feeding behaviors [32]. Thus, increased feeding behavior in the open field test may suggest a lower level of fear in birds in the Ba_CP group. Freezing duration has been shown to be positively correlated with fear and anxiety when animals experience stress [33]. Mice with a high rate of anxiety-like behaviors had longer freezing times compared to control mice. Notably, the anxiety-like behavior was further reduced by the treatment of Nepicastat [34]. In this study, the duration of freezing was significantly lower in birds supplemented with BaSC06 than in CP birds. We did not have direct evidence that BaSC06 may play a role in alleviating fear and stress-related behaviors, and further study is needed to classify their relationship. Additionally, CP-infected birds showed more aggressive and feather-pecking behaviors, which were lower in the BaSC06 group. Aggressive behavior is known to be associated with stress [35], and stress can also induce feather pecking in layer hens [36]. As mentioned in the introduction, probiotics are known to improve normal behaviors in mice [15]. In this study, BaSC06 is probably associated with the reduction of stress- and fear related behaviors in broilers, and the underpinning mechanism requires further investigation.

*HTR1A* is involved in the regulation of a variety of neurotransmitters and hormones, and it is associated with aggressive, feather-pecking, and toe-pecking behaviors in chimpanzees [37]. In this study, aggressive and feather-pecking behavior as well as the relative expression of *HTR1A* were significantly higher in CP-infected birds than those in the other three groups at 50 days of age. These results were consistent with a previous study showing that layer hens with higher pecking behaviors showed a higher expression of *HTR1A* [38]. Feather pecking and aggressive behaviors decreased after the treatment of BaSC06. Further, the lower expression of *HTR1A* in Ba_CP birds than in CP birds provides evidence of the possible inhibitory effect of BsSC06 on aggressive and feather-pecking-related gene expression of *HTR1A*. *GABA* has a postsynaptic inhibitory effect, which is closely related to the promotion of glucose metabolism, anti-anxiety, and stress, as well as improved brain function. In this study, *GABA* expression in the Ba group was significantly higher than that in the CP group and the Ba_CP group, indicating that BaSC06 can reduce *GABA* expression. *GABRB1* is closely related to the development of the central nervous system, synaptic composition, synaptic transmission, and neurotransmitter receptor activity, as well as stress, affective disorder, cognitive function, learning, and memory ability [39,40]. Central nervous system dysfunction is known to lead to the downregulation of *GABRB1* expression [41]. The absence or imbalance of *GABRB1* can lead to the damage of neuronal cells and behavioral and cognitive dysfunction in animals [39]. In this study, the expression of *GABRB1* in the Ba_CP group was significantly higher than that in the CP group at the age of 50 days, indicating that BaSC06 may probably promote the expression of *GABRB1* in the hypothalamus, which may be associated with its contribution to the development of the nervous system. Recently, increased studies indicated that probiotics modulate the pecking behaviors in layers, which is probably through the role of the gut–brain axis [21,22,23]. We speculated that the protective effects of BaSC06 on behaviors and brain functions may be associated with the role of the gut–brain axis in the current study. Investigation is needed to further classify the underpinning mechanism of probiotics via the role of the gut–brain axis.

The enterotoxin produced by CP is known to be able to cross the blood–brain barrier, damage the vascular system, and cause swelling, vacuolization, and necrosis of endothelial cells [42]. CP can cause brain vacuolation and mild edema, loose, tight connections in the cerebral vascular endothelium, swelling and rupture of perivascular astrocytes, elevated intracerebral pressure, focal to diffuse degeneration and necrosis of the bisymmetric region, and even encephalomalacia in mice [43,44]. On the other hand, probiotics are known to modulate gut and brain functions involved in the digestion and conversion of food materials into many useful substrates for the host [45]. *Lactobacillus* plantarum-derived postbiotics markedly suppressed brain injuries and neuroinflammation and effectively prevented *Salmonella enterica Typhimurium* infection in mice [46]. In this study, CP-infected birds had thickened pia mater, a loose structure of the cerebral cortex, edema of some neurons, and serious cell vacuolization, similarly to the mice mentioned above. Along with the infection period, the structure of the pia mater became loose, and the cerebral cortex showed microvascular bleeding, vasodilation, edema, and increased vascular sheathing, indicating that the infection of CP could cause obvious pathological damage to the brain broilers. The longer the infection period, the more serious the symptoms. Notably, brain damage and pathology were impaired in the treatment of the BaSC06 group, indicating the preventive effects of BaSC06 on the pathological damage to the pia and cortex of the brain against CP infection.

In this study, nervous system and disease-related genes were identified. Likewise, in the comparison of the control and the CP group, the significantly upregulated *LRATD2* is the main inducer of neuroendocrine tumor, which induces glioma by the cell cycle or Akt/GSK-3β/βpathways [47,48,49]. Significantly upregulated *RSAD2* is involved in the process of spinal cord injury and is associated with the staging, grading, and lymphatic metastasis of malignant tumors [50,51], while *SLC44A2* is associated with ventricular tachycardia and the formation of venous thrombosis [52]. Chickens infected with reovirus and Newcastle disease virus showed significant upregulation of *IFIT5* [53,54], which was also seen to be upregulated in the comparison between the control and Ba groups. Gene *PRRX2* can promote the drug resistance of advanced prostate cancer and the malignant phenotype of adenocarcinoma of the lung, while the knockout of *PRRX2* can effectively inhibit the proliferation and growth of breast cancer [55,56], which was seen to be downregulated in the comparison between the control and Ba groups. In the comparison of the CP and Ba groups, the upregulated gene *ACVR1C* is known to promote the proliferation of retinoblastoma, increase the prevalence of type II diabetes, and reduce fat content [57,58]. Gene *NPSR1* promotes the malignant phenotype of adenocarcinoma of the lung and thyroid cancer cells [59], and *Ano2* is associated with multiple sclerosis [60]. While the downregulated gene *OSGEP* is associated with tRNA modification, and its mutations can lead to various neurological abnormalities [61], *SMKR1* is involved in the process of cancer occurrence, which is closely related to cancer recurrence. Accordingly, the CP infection is associated with the upregulated expression of disease-related genes, including *LRATD2, RSAD2, IFIT5,* and *PRRX2*, while the treatment is linked to the downregulate expression of disease-related genes, including *ACVR1C, NPSR1, Ano2, OSGE,* and *SMKR1.*

Between the control and CP groups, differently expressed genes were enriched in three pathways related to disease pathways. These diseases are reported to cause disfunction of the brain, liver, and neurons [62,63]. The differently expressed genes between the CP and Ba groups were enriched into similar pathways as the above comparison, of which most were of disease relevance. In the comparison of CP and Ba_CP groups, the upregulated gene *OPN3* is associated with the development of the central and peripheral nervous system, which modulates various motor and sensory systems, memory, and emotions [64], and *TMEM213* is to improve the treatment of lung adenocarmosis and promote the positive effect of drugs on the organism [65]. The downregulated gene *Adam32* is positively correlated with the occurrence of hepatoblastoma, and it is known to promote cancer stem cell and epithelial mesenchymal transformation and induce cancer [66]. The downregulated gene *RAB17* is associated with the increase in drug resistance in ovarian cancer and the promotion of the proliferation of cancer cells [67]. In the comparison of the control vs. Ba_CP group, the upregulated gene *GPR12* in the hypothalamus is closely related to short-term memory, which plays a major role in neurogenesis and neuroinflammation [68]. In addition, *BF2* acts to inhibit the immune response of antigen-specific T lymphocytes [69]. The enriched pathways were similar among the three comparisons, including control vs. Ba_CP, Ba vs. Ba_CP, and CP vs. Ba_CP. The pathway of longevity regulation is closely related to insulin secretion, energy metabolism regulation, and oncogenes [70]. Stress and neurodegenerative diseases would induce the upregulation of autophagy pathways, which is necessary to maintain homeostasis and prevent oxidative stress and other damage [71]. Spinocerebellar ataxia is caused by degenerative degeneration and atrophy of the cerebellum, brain stem, and spinal cord in animals, and the affected individuals show symptoms such as limb disharmony, dementia, and distal muscular atrophy [72]. Cholinergic, glutamate synapse, and dopaminergic synapse are significantly related to learning, memory, cognitive function, motor function, and emotion, whereas the dysfunction of these pathways can cause anxiety, depression, Alzheimer’s disease, epilepsy, and other nervous system diseases [73,74,75]. Growth hormone can promote bone, visceral, and body growth, promote protein synthesis, participate in fat and mineral metabolism, and play a crucial role in growth and development [76]. In relation to the KEGG pathways, disease relevance pathways are found, referring to the comparison with the CP infection group. Notably, pathways are mainly regarding the nervous development pathways when referring to the treatment of the BaSC06 group, such as longevity regulation, cholinergic, glutamate synapse, and dopaminergic synapse pathways.

In summary, our study provides an insight into the possible role of BaSC06 on behaviors, related gene expression, and brain functions; however, there are still limitations. The underpinning mechanism of BaSC06 on behavior and gene expression needs to be identified, which may be achieved by microbial transplantation.

## 5. Conclusions

In conclusion, CP increased fear behavior, inhibited the expression of *GABA,* and increased the expression of *HTR1A* in the hypothalamus of broilers. CP caused obvious pathological damage to the pia mater and cortex of the brain. Treatment with probiotic BaSC06 is probably linked to reduced fear behaviors and the promotion of the expression of the anti-anxiety genes *GABA* and *GABRB1*. Additionally, BaSC06 exerted protective roles on the pia mater and cortex of the brain. We consider that treatment with BaSC06 may improve brain function by inhibiting the expression of disease-related genes, promoting the activity of genes related to nervous system development, and activating pathways related to energy metabolism and nervous system development.

## Figures and Tables

**Table 1:** The top five significantly different genes and enriched KEGG pathways between the comparison groups.

Comparable Group	Upregulated Gene Name	Down-Regulated Gene Name	Pathways
Control VS. CP	LRAT domain containing 2 Radical S-adenosyl methionine domain containing 2 Solute carrier family 44 member 2 Interferon induced protein with tetratricopeptide repeats 5	Paired related homeobox 2	Choline metabolism in cancer, Hepatitis C, and Influenza A
Control VS. Ba_CP	Activin A receptor type I G protein-coupled receptor 12	Osialoglyco protein endopeptidase	Longevity regulating pathway–mammal, Autophagy–animal, Longevity regulating pathway–multiple species, GnRH secretion, Spinocerebellar ataxia, Cholinergic synapse, Glutamatergic synapse, Growth hormone synthesis, secretion and action
Ba VS. CP	Major histocompatibility complex class I antigen BF2	/	Type I diabetes mellitus, Allograft rejection, Autoimmune thyroid disease, Viral myocarditis, Cellular senescence, Kaposi sarcoma-associated herpesvirus infection, and Viral carcinogenesis
Ba VS. Ba_CP	Activin A receptor type 1C Neuropeptide S receptor 1 Anoctamin 2	Osialoglyco protein endopeptidase Small lysine rich protein 1	Autophagy–animal, Longevity regulating pathway–mammal, cholinergic synapse, Glutamatergic synapse, Longevity regulating pathway–multiple species, Dopaminergic synapse, and GnRH secretion
CP VS. Ba_CP	Opsin 3 Transmembrane protein 213	O-sialoglyco protein endopeptidase A disintegrin and metalloproteinase domain 32 Member RAS oncogene family	Cholinergic synapse, Spinocerebellar ataxia, Glutamatergic synapse, cAMP signaling pathway, GnRH secretion, Dopaminergic synapse, Circadian entrainment, Insulin secretion, Growth hormone synthesis, and secretion and action

Note: Control is the control group; Ba is *Bacillus amyloliquefaciens* SC06 group; CP is *Clostridium perfringens* group; and Ba_CP is *Bacillus amyloliquefaciens* SC06 plus *Clostridium perfringens* group.

## Data Availability

Data are contained within the article and Supplementary Materials.

## Supplementary Materials

The following are available online at https://www.mdpi.com/article/10.3390/ani14111547/s1. Figure S1. Structural changes in the left side of the brain in broilers 21 days of age in response to hematoxylin–eosin staining, Figure S2. Structural changes in left side brain of broilers at the age of 50 days in response to hematoxylin—eosin staining, Figure S3. Validation of differential mRNAs expression by RT-qPCR, Table S1. The main content of the diet, Table S2. The definition of behaviors, Table S3. Primers of genes, Table S4. Sequencing data quality, Table S5. Contrast with chicken genomes.
