# Supplementary material for: The Effects of Bacillus amyloliquefaciens SC06 on Behavior and Brain Function in Broilers Infected by Clostridium perfringens
Source: Animals (Basel). 2024 May 23;14(11):1547. doi: 10.3390/ani14111547 (PMC11171150; doi:10.3390/ani14111547)
Supplement: Supplementary file 1 [file animals-14-01547-s001.zip › animals-2958796-supplementary.pdf]

Effects of *Bacillus amyloliquefaciens* SC06 on behavior and brain function in broilers infected by *Clostridium perfringens*

Siyu Chen[1], Jinling Liu[1], Shuyan Luo[1], Limin Xing[1], Li Gong[1,2], Weifen Li[2]

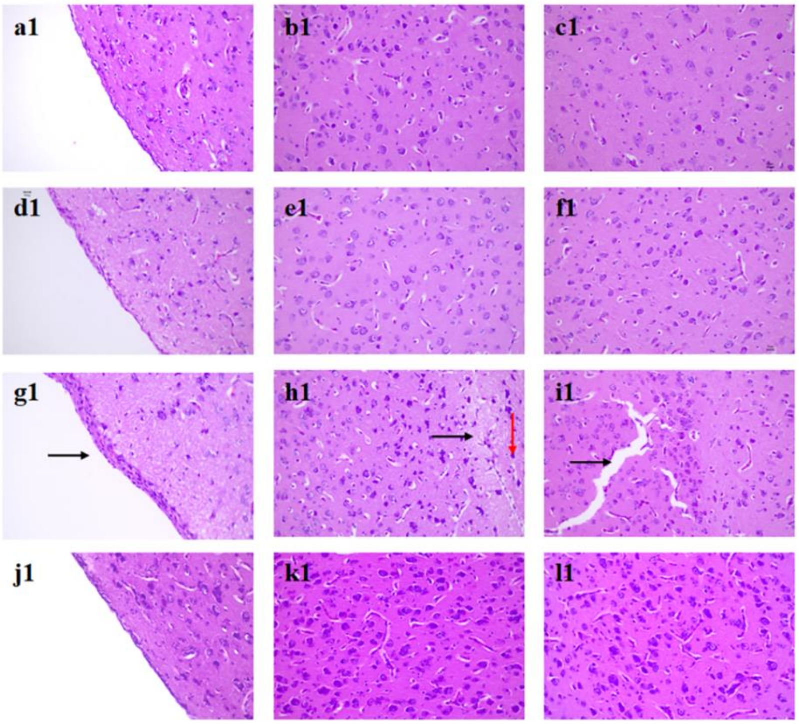

Figure S1. Structural changes of the left side of the brain in broilers 21 days of age in response to hematoxylin-eosin staining. a1, b1 and c1 are control group (Control group), d1, e1 and f1 are group Ba (*Bacillus amyloliquefaciens* SC06 group), g1, h1 and i1 are group CP (*Clostridium perfringens* group), j1, k1 and l1 are group Ba_CP (*Bacillus amyloliquefaciens* SC06 plus *Clostridium perfringens* group).

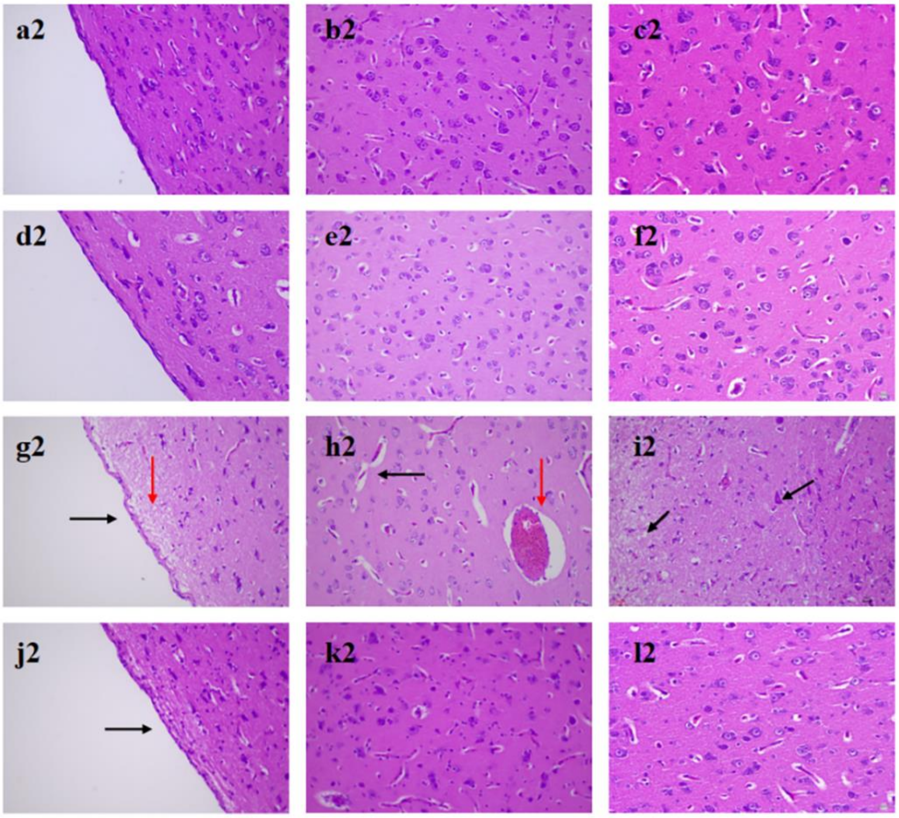

Figure S2. Structural changes of left side brain of broilers at the age of 50 days in response to hematoxylin-eosin staining. a2, b2 and c2 are control group (Control group), d2, e2 and f2 are group Ba (*Bacillus amyloliquefaciens* SC06 group), g2, h2 and i2 are group CP (*Clostridium perfringens* group), j2, k2 and l2 are group Ba_CP (*Bacillus amyloliquefaciens* SC06 plus *Clostridium perfringens* group).

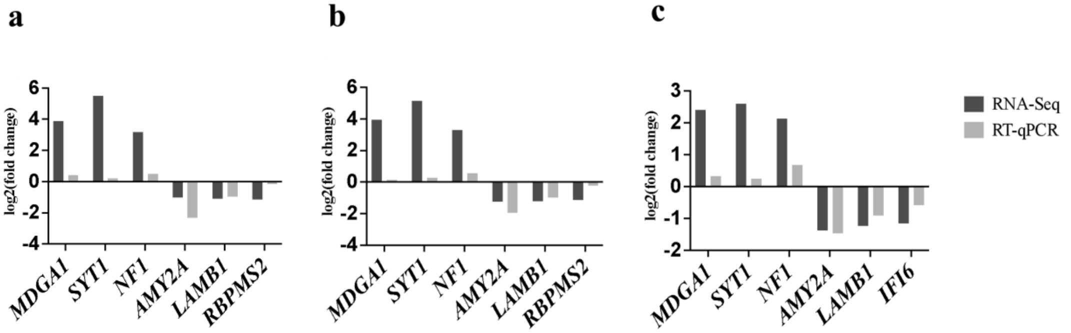

Figure S3. Validation of differentially mRNAs expression by RT-qPCR. (a) Differently expressed genes in the comparison of Control vs Ba_CP; (b) Differently expressed genes in the comparison of Ba vs Ba_CP; (c) Differently expressed genes in the comparison of CP vs Ba_CP.

Table S1. Basic diet ingredients and nutritional composition.

Raw material	Starter (1–20 Days)	Finisher (21–50 Days)
Ingredients (g/kg)		
Corn	58.75	57.66
Soybean meal (43% CP)	27.70	27.21
Corn gluten meal (60% CP)	5.00	5.00
Limestone	3.00	
Calcium hydrogen phosphate (16.5%)	1.43	1.22
L-lysine Sulfate (70%)	1.10	1.04
DL-methionine (98.5%)	0.66	0.39
NaCl	0.33	0.21
L-Threonine	0.28	0.32
Peanut meal	0.16	0.06
Choline chloride (50%)	0.08	0.08
Premix 1	0.35	0.35
Phytase (20,000 IU)	0.01	0.01
Sodium humate	0	0.15
Lard	1.15	6.30
Total (%)	100	100
Nutrient content 2		
ME (Kcal/kg)	2941.61	3229.20
CP (g/kg)	21.92	19.61
CEE (g/kg)	3.65	8.87
CF (g/kg)	2.51	1.53
Ca (g/kg)	0.90	0.80
Total P (%)	0.57	0.54
Lys (%)	1.48	1.21
Met (%)	0.64	0.52
Cys (%)	0.30	0.27
Met + Cys (%)	0.94	0.78
L-Threonine	0.92	0.79

1 Premix is provided for each kilogram of diet: vitamin A, 12,000 IU; vitamin D3, 3000 IU; vitamin E, 10 IU; vitamin K3, 2 mg; vitamin B1, 1 mg; vitamin B2, 3 mg; vitamin B6, 2 mg; vitamin B12, 0.01 mg; niacin, 20 mg; calcium pantothenate, 4 mg; biotin, 0.05 mg; folic acid, 0.5 mg; Mn, 100 mg; Fe, 100 mg; Zn, 80 mg; Cu, 20 mg; I, 3 mg; and Se, 0.5 mg. 2 Nutrient levels were calculated values. L-, L-isomer; DL-, DL-isomer.

Table S2. The definition of Behaviors

Behavior variable	Behavior description
Exploration	Pecking or scratching the ground/environment using claws or beak or non-aggressively pecking at another hen's feathers.
Feeding	Feeding diets
Lay-resting	Lying immobile with eyes open or closed.
Flapping	Non-aggressive locomotion of flapping its wings at a normal or faster than normal pace.
Stand-resting	Standing immobile with eyes open or closed.
Preening	Using the beak or claws to rub or comb feathers.
Aggressive	Attacking another bird with the beak or claws either by prolonged episodes of pecking and scratching or continuous performance of this behavior.
Toe-picking	Attacking another bird's toe with the beak or claws either by prolonged episodes of pecking and scratching or continuous performance of this behavior.
Feather pecking	Aggressively pecking the feathers of another bird with the beak for only a fraction of a second. Every aggressive peck was recorded as a separate event.

Table S3. Primers of genes

Gene	Primer name	Sequences (5′-3′)
GAPDH	GAPDH-F	TATCTTCCAGGAGCGTGACC
	GAPDH-R	AGCACCACCCTTCAGATGAG
HTR1A	HTR1A-F	GGTGCTGAACAAGTGGACTCTG
	HTR1A-R	AAGAAGCCGATGAGCCAGGT
DA	DA-F	GCTTGTGGAAAGGGACTCT
	DA -R	GGCGAAACCTAATGACAGC
GABA	GABA-F	GGCGGTCGGAATATCGGAAT
	GABA-R	TTCATGGTGGGCTCTGGAAC
GABRB1	GABRB1-F	TGATTCGGCTCCATCCTGAT
	GABRB1-R	CTGACCAGTCTCTGCTCGAT

Note: *HTR1A*: 5-hydroxytryptamine receptor 1A, DA: dopamine, *GABA*: Gamma-aminobutyric acid, and *GABRB1*: Gamma-aminobutyric acid type A receptor subunit beta1.

Table S4. Sequencing data quality

Sample name	Original data	Valid data (%)	The total number of data bases (bp)	Total number of filtered high quality data bases(bp)	Q30 (%)	GC content (%)
Control1	46556390	46250738 (99.34%)	6983458500	6898111091	92.34	46.86
Control2	40367716	40113050 (99.37%)	6055157400	5985038025	92.75	47.02
Control3	42537096	42266186 (99.36%)	6380564400	6303160851	93.18	46.91
Control4	43531296	43265596 (99.39%)	6529694400	6454341967	92.57	46.92
Control5	42318516	42063158 (99.40%)	6347777400	6270941217	92.96	46.04
Ba1	39906558	39644288 (99.34%)	5985983700	5919051774	92.85	47.15
Ba2	46637354	46348670 (99.38%)	6995603100	6915735566	92.59	47.38
Ba3	45435246	45135674 (99.34%)	6815286900	6733307772	92.82	46.94
Ba4	44017682	43716166 (99.32%)	6602652300	6518486563	92.79	46.89
Ba5	40222936	39965528 (99.36%)	6033440400	5961112114	93.29	47.14

Ba6	36733840	36511418 (99.39%)	5510076000	5446519338	92.75	46.4
CP1	38145560	37877002 (99.30%)	5721834000	5651031867	92.98	47.02
CP2	41008186	40741162 (99.35%)	6151227900	6068989171	92.52	47.16
CP3	37781060	37553260 (99.40%)	5667159000	5595374784	92.31	46.73
CP4	37435684	37236084 (99.47%)	5615352600	5559784671	93.36	46.29
CP5	42423006	42212416 (99.50%)	6363450900	6295181498	93.49	46.65
CP6	36965482	36787290 (99.52%)	5544822300	5490808228	93.28	46.66
Ba_CP1	40210996	40020702 (99.53%)	6031649400	5971375258	93.65	46.73
Ba_CP2	39822112	39631968 (99.52%)	5973316800	5920150897	93.15	47.06
Ba_CP3	44269670	44061464 (99.53%)	6640450500	6579880272	93.50	47.1
Ba_CP4	45395548	45188430 (99.54%)	6809332200	6748624928	93.61	47.57
Ba_CP5	44184008	43971670 (99.52%)	6627601200	6565621276	93.30	47.68

Note: Control is Control group, Ba is *Bacillus amyloliquefaciens* SC06 group, CP is *Clostridium perfringens* group, and Ba_CP is *Bacillus amyloliquefaciens* SC06 plus *Clostridium perfringens* group.

Table S5. Contrast with chicken genomes

Sample name	Go to rRNA reads	The ribosome reads of this species were compared	Located in the chicken genome (%)	Exon region proportion
Control1	44488260	1762478	94.63	38496885 (91.45%)
Control2	38621550	1491500	93.53	32656701 (90.40%)
Control3	40371936	1894250	94.60	34075797 (89.22%)
Control4	41225274	2040322	94.31	34924255 (89.83%)
Control5	40645150	1418008	94.33	32579367 (84.98%)
Control6	39969030	1576708	94.68	35618768 (94. 12%)
Ba1	38233378	1410910	93.71	32799058 (91 54%)
Ba2	44482100	1866570	94.46	39320213 (93 58%)
Ba3	43338944	1796730	93.39	36746970 (90 79%)
Ba4	42250474	1465692	94.00	34659601 (87.27%)
Ba5	38473554	1491974	94.49	33395152 (91.8%)
Ba6	35260726	1250692	93.83	28043042 (84.76%)
CP1	36481404	1395598	94.27	30469098 (88.60%)
CP2	38991576	1749586	94.44	33308913 (90.46%)
CP3	36047706	1505554	94.22	29636267 (87.25%)
CP4	36261320	974764	94.83	28295521 (82.29%)
CP5	41180440	1031976	94.49	31315914 (80.48%)
CP6	36504612	282678	94.26	25365086 (73 72%)
Ba_CP1	39679240	341462	94.36	27902894 (74.52%)
Ba_CP2	39338622	293346	93.46	27527818 (74.87%)
Ba_CP3	43624822	436642	93.80	30885597 (75.48%)

Ba_CP4	44893956	294474	93.86	31987591 (75.91%)
Ba_CP5	43654248	317422	93.45	31146482 (76.35%)

Note: Control is Control group, Ba is *Bacillus amyloliquefaciens* SC06 group, CP is *Clostridium perfringens* group, and Ba_CP is *Bacillus amyloliquefaciens* SC06 plus *Clostridium perfringens* group.
